# Deployment, dispatch, and delivery: a scoping review of drone-delivered AED for out-of-hospital cardiac arrest

**DOI:** 10.3389/fpubh.2026.1839209

**Published:** 2026-05-29

**Authors:** Pengfei Cheng, Baichao Xu, Hua Zhang, Haizhen Wang, Ying Xiao

**Affiliations:** 1 Faculty of Medicine, Macau University of Science and Technology, Macau, China; 2 Division of Sports Science and Physical Education, Tsinghua University, Beijing, China; 3 Department of Physical Education, Hainan Medical University, Haikou, China; 4 School of Nursing, Hainan Medical University, Haikou, China; 5 Hainan Health Vocational College, Haikou, China; 6 Department of Emergency Medicine, The Second Affiliated Hospital Zhejiang University School of Medicine, Hangzhou, China

**Keywords:** automated external defibrillator, emergency medical services, out-of-hospital cardiac arrest, scoping review, unmanned aerial vehicle

## Abstract

**Objective:**

Out-of-hospital cardiac arrest (OHCA) constitutes a significant global public health burden, and early defibrillation is essential for patient survival. However, traditional emergency medical services frequently struggle to achieve the requisite response times. Drone-delivered automated external defibrillators (AEDs) have emerged as an innovative intervention strategy to address this challenge. This scoping review aimed to systematically map the current evidence on drone-delivered AED systems for OHCA and identify knowledge gaps for future research.

**Methods:**

Systematic searches were conducted across PubMed, Embase, Web of Science, Cochrane Library, Scopus, CINAHL, and IEEE Xplore databases. Eligible studies were analyzed using an inductive coding approach, and findings were synthesized into a conceptual framework.

**Results:**

Eighteen studies were included. Evidence was organized into a “3D Framework” comprising three operational dimensions: Deployment (where), Dispatch (when), and Delivery (how). Multi-rotor drone systems demonstrated preliminary operational readiness with high delivery success rates (81–100%) with no reported injuries or serious adverse safety events. Time advantages over conventional emergency medical services were context-dependent, with drones arriving first in 67–93% of rural cases versus 32% in urban settings. One successful case of drone-delivered AED defibrillation with patient survival has been documented.

**Conclusions:**

Current evidence, predominantly from simulation studies, suggests that drone-delivered AED systems demonstrate preliminary technical feasibility and potential time advantages, particularly in rural and underserved areas. However, real-world clinical evidence remains limited, and findings should be interpreted with caution given language restrictions, study heterogeneity, and limited geographic and socioeconomic diversity of included studies. Further research is needed to validate these preliminary findings.

## 1 Introduction

Out-of-hospital cardiac arrest (OHCA) is a sudden and highly fatal medical emergency that constitutes one of the most significant global public health challenges. It is estimated that more than 3.5 million OHCA cases occur worldwide annually, with overall prehospital survival rates persistently below 10% (1). Substantial variations exist among countries in both incidence and survival rates, and neurological outcomes remain universally poor. According to the American Heart Association's 2024 report, the incidence of OHCA in the United States is 88.8 per 100,000 population, with a survival-to-discharge rate of only 9.3% and a mere 7.5% of survivors achieving favorable neurological function (2). In Europe, the average incidence is 55 per 100,000 population, with an average survival rate of 7.5% (3). In China, the OHCA incidence reaches 95.7 per 100,000 population (4); however, the survival-to-discharge rate ranges from only 1.2% to 2.8%, with just 0.8% of survivors demonstrating favorable neurological outcomes at discharge (4). These data reveal substantial disparities across healthcare systems and underscore the urgent need to improve OHCA outcomes.

Successful resuscitation of OHCA patients depends on each link in the chain of survival, which includes recognition of cardiac arrest and activation of the emergency response system, early cardiopulmonary resuscitation (CPR), rapid defibrillation, advanced resuscitation by Emergency Medical Services (EMS) and other healthcare providers, post-cardiac arrest care, and recovery (5). Among these, early out-of-hospital defibrillation using an automated external defibrillator (AED) is considered a critical determinant of patient survival (5). Evidence indicates that approximately 53% of OHCA cases present with shockable rhythms, and if defibrillation is administered within the golden 4 min, survival rates can reach 50% to 70%; otherwise, survival probability decreases by 7% to 10% with each passing minute (6). However, due to the uneven distribution of EMS resources, constrained dispatch routes, and traffic congestion, EMS response times frequently fail to meet the time-critical defibrillation requirements, representing a primary point of failure in the OHCA chain of survival (7, 8).

To address the prehospital defibrillation challenges facing OHCA patients and to increase prehospital defibrillation rates while reducing time to first shock, international organizations including the American Heart Association, European Resuscitation Council, and International Liaison Committee on Resuscitation have advocated for the global implementation of Public Access Defibrillation (PAD) programs, which involve the widespread deployment of AEDs in public spaces (3, 5). Nevertheless, significant challenges persist, including low AED deployment density, extensive coverage gaps, and insufficient public awareness and utilization rates (9, 10). Statistical data indicate that globally, fewer than 2% to 37.4% of OHCA patients receive AED defibrillation prior to EMS arrival (11). Furthermore, approximately 70% of OHCA events occur in private settings such as homes, where timely access to nearby public AEDs is difficult (11). These accessibility issues are particularly pronounced in rural areas, remote regions, and urban areas with complex traffic conditions (8, 12). Consequently, there is an urgent need to explore novel, efficient, and mobile prehospital defibrillation solutions capable of reducing response times and extending coverage.

With the maturation of unmanned aerial vehicle technology and declining costs, the healthcare sector has progressively explored drone applications in the transport of blood products, vaccines, and medications (13). Drone-based blood delivery programs in multiple countries have achieved routine operations, significantly improving blood supply accessibility in remote areas (14). During the COVID-19 pandemic, the use of drones to transport diagnostic samples and vaccines further validated the value of this technology in public health emergency response (15). Given the decisive role of rapid response in improving OHCA patient outcomes, drone-delivered AEDs have emerged as a novel intervention capable of overcoming ground traffic limitations and PAD coverage gaps, garnering considerable attention in the field of emergency medicine (16). Drones can fly directly to destinations via linear paths unimpeded by ground traffic conditions, enabling precise AED delivery to patient locations within minimal timeframes and providing OHCA patients with opportunities for early out-of-hospital defibrillation (17). In rural and remote areas where EMS response times are prolonged, the time advantages of drone-delivered AEDs may be particularly pronounced, potentially filling coverage gaps left by traditional emergency medical systems (18). Additionally, drone systems can maintain round-the-clock readiness and rapid deployment capabilities, unconstrained by personnel scheduling and vehicle dispatch limitations, potentially demonstrating unique flexibility in multiple-casualty incidents or large-scale emergency scenarios (19).

In recent years, research on drone-delivered AEDs has proliferated, progressing from early computational simulation studies validating technical feasibility to pilot implementations in actual OHCA scenarios (18). However, systematic evidence synthesis addressing the most recent developments in this field remains lacking. Existing studies exhibit considerable heterogeneity in study design, geographic settings, technical parameters, and outcome measures, necessitating clarification of the applicability and comparability of research findings. Moreover, the translation of this technology from laboratory settings to clinical application continues to face numerous challenges, requiring comprehensive updating and analysis based on current evidence. Therefore, this scoping review was conducted to systematically map the current evidence on drone-delivered AED systems for OHCA response. Guided by the Population-Concept-Context (PCC) framework (20), this review addressed three key questions concerning: (1) deployment strategies, dispatch integration, and flight parameters of drone-delivered AED systems; (2) time advantages compared to conventional emergency response; and (3) delivery success rates, operational feasibility, safety, and patient outcomes across diverse settings.

## 2 Methods

This study was conducted following the methodological framework for scoping reviews proposed by Arksey and O'Malley (21), with refinements incorporated from the updated Joanna Briggs Institute guidance for scoping reviews by Peters et al. (22). The reporting of this review adheres to the Preferred Reporting Items for Systematic Reviews and Meta-Analyses extension for Scoping Reviews (PRISMA-ScR) guidelines (23); the PRISMA-ScR checklist is provided in Supplementary material. The study protocol was prospectively registered on the Open Science Framework (Registration 10.17605/OSF.IO/CQXWT).

### 2.1 Identification of research questions

The scope of this scoping review was delineated using the PCC framework (20), with each element defined as follows: the population included actual OHCA patients, individuals highly suspected of experiencing OHCA, or simulated OHCA patients within scenario-based experimental designs; the concept pertained to the delivery of automated external defibrillators via unmanned aerial vehicles; and the context incorporated both real-world and simulated environments across diverse geographic settings, including urban, rural, suburban, and mountainous areas.

Guided by this framework, the present scoping review sought to address the following research questions: (1) What deployment strategies, dispatch integration methods, and drone flight parameters have been utilized in drone-delivered AED systems for OHCA response? (2) What is the evidence regarding the time advantage of drone-delivered AED compared to conventional EMS or bystander-retrieved AED during the prehospital phase of OHCA? (3) What is the evidence regarding delivery success rates, operational feasibility, safety, and patient outcomes across diverse application settings?

### 2.2 Search strategy

A systematic search was conducted across the following electronic databases: PubMed, Embase, Web of Science, Cochrane Library, Scopus, CINAHL, and IEEE Xplore. The search timeframe spanned from database inception to March 20, 2026. The search strategy was developed using a combination of MeSH terms and free-text keywords, structured around three core concepts: (1) unmanned aerial vehicle-related terms; (2) automated external defibrillator-related terms; and (3) out-of-hospital cardiac arrest-related terms. Search terms within each concept were combined using the Boolean operator “OR”, while concepts were linked using “AND”. The search strategy was formulated by a researcher with systematic review expertise and subsequently verified through team discussion.

The PubMed search strategy was as follows:((“Unmanned Aerial Devices” [MeSH Terms]) OR (drone^*^ [Title/Abstract]) OR (unmanned aerial vehicle^*^ [Title/Abstract]) OR (UAV [Title/Abstract]) OR (unmanned aircraft system^*^ [Title/Abstract]) OR (UAS [Title/Abstract]) OR (remotely piloted aircraft^*^ [Title/Abstract])) AND ((“Defibrillators” [MeSH Terms]) OR (automated external defibrillator^*^ [Title/Abstract]) OR (automatic external defibrillator^*^ [Title/Abstract]) OR (AED [Title/Abstract]) OR (defibrillator^*^ [Title/Abstract]) OR (defibrillation [Title/Abstract])) AND ((“Out-of-Hospital Cardiac Arrest” [MeSH Terms]) OR (out-of-hospital cardiac arrest^*^ [Title/Abstract]) OR (OHCA [Title/Abstract]) OR (out of hospital cardiac arrest [Title/Abstract]) OR (sudden cardiac arrest^*^ [Title/Abstract]) OR (sudden cardiac death [Title/Abstract]) OR (cardiac arrest [Title/Abstract])).

Search strategies for other databases were adapted according to their respective syntax rules and thesauri, as detailed in Supplementary material. Furthermore, to ensure comprehensive literature retrieval, the following supplementary search methods were employed: (1) searching Google Scholar; (2) searching the gray literature database OpenGrey; (3) manually screening the reference lists of all included studies; and (4) forward citation tracking of included studies.

### 2.3 Eligibility criteria

The inclusion criteria were defined according to the PCC framework: (1) Population: real OHCA patients, highly suspected OHCA patients, or simulated OHCA patients; (2) Concept: studies examining the design, testing, implementation, or evaluation of drone-delivered AEDs; (3) Context: real-world or simulated environments without geographic restrictions; (4) Study design: randomized controlled trials, non-randomized controlled trials, before-and-after studies, simulation studies involving actual drone flights, feasibility studies, observational studies, and case studies; and (5) Outcomes: studies reporting at least one of the following: response time, delivery success rate, proportion of drone-first arrivals, time to first defibrillation, injuries or serious adverse safety events, or other drone-delivered AED performance metrics.

Studies were excluded if they: (1) relied exclusively on theoretical modeling, mathematical simulation, or geographic information system (GIS)-based spatial analysis without real-world or scenario-based drone flight components; (2) were conference abstracts, editorials, commentaries, or reviews; (3) had unobtainable full texts; (4) were duplicate publications (for multiple reports from the same study, only the version with the most complete data or most recent publication was retained); (5) were published in languages other than Chinese or English; or (6) were non-peer-reviewed or lacked original research data.

It is important to clarify the distinction between “simulation studies” included in this review and the excluded “mathematical modeling/GIS-based analyses.” In this review, simulation studies refer to investigations involving actual drone flights, physical AED deliveries, or realistic scenario-based tests conducted in real-world or controlled environments, where operational performance can be empirically observed and measured. In contrast, mathematical modeling and GIS-based spatial analyses rely on theoretical calculations and computational optimization based on assumed parameters (such as hypothetical flight speeds, coverage radii, or cardiac arrest distributions), without empirical validation through actual drone operations. While such modeling approaches are valuable for informing deployment strategies and network optimization, they cannot fully capture real-world operational challenges including adverse weather conditions, technical malfunctions, communication failures, and human factors. As drone-delivered AED systems are now transitioning from theoretical concept to clinical implementation in several countries, this review intentionally focused on empirical evidence from actual flight operations to provide a realistic assessment of current capabilities, limitations, and evidence gaps.

### 2.4 Study selection

Retrieved records were imported into EndNote 21 for management, with duplicates removed through automatic deduplication followed by manual verification. Two trained reviewers (PF.C & H.Z) independently conducted study selection using a two-stage process. During title and abstract screening, records were categorized as “include,” “exclude,” or “uncertain” based on predetermined eligibility criteria. Subsequently, full texts were obtained for records marked as “include” or “uncertain” and assessed for final eligibility. For inaccessible full texts, interlibrary loan requests and author contact were attempted; persistently unavailable studies were excluded with reasons documented.

Reviewers compared results after each stage, resolving disagreements through discussion or, when necessary, arbitration by a third reviewer (BC.X). Excluded studies and exclusion reasons were recorded throughout. The selection process and record numbers at each stage are illustrated in a PRISMA flow diagram.

### 2.5 Data extraction

Data extraction was performed independently by two reviewers (PC.F & HZ.W) using a standardized form developed based on the PCC framework and JBI scoping review guidelines (22). The form was pilot-tested on two to three randomly selected studies and refined accordingly to ensure applicability and consistency in interpretation. The reviewers cross-checked extraction results, resolving discrepancies through discussion or consultation of original publications. Authors were contacted when information remained unclear. For studies with multiple publications, data were consolidated to maximize completeness. Any assumptions or simplifications during extraction were documented.

Extracted data included: (1) Publication characteristics: first author, publication year, and country/region; (2) Study characteristics: design, objectives, setting (urban, rural, suburban, mountainous), and sample size or simulation runs; (3) Population characteristics: participant type (real OHCA patients or simulated scenarios); (4) Drone system characteristics: drone type and model, flight performance parameters (maximum speed, distance, and altitude), payload capacity, AED type and model, and delivery method; (5) Implementation characteristics: deployment model (fixed base, mobile, or EMS integration) and dispatch system integration; and (6) Outcomes:response time (definition and measurement), delivery success rate and failure reasons, proportion of drone-first arrivals, bystander interaction, patient outcomes, injuries or serious adverse safety events, and other reported outcomes.

### 2.6 Data synthesis

Data were synthesized using a narrative descriptive approach following established scoping review methodology. Key characteristics of included studies were summarized in tabular format, including study design, setting, drone specifications, delivery methods, and primary outcomes.

Thematic synthesis was conducted using a three-phase inductive coding approach to develop a conceptual framework. In the initial coding phase, two reviewers (PC.F & HZ.W) independently read and coded the full texts of all included studies, extracting key concepts, operational characteristics, performance metrics, and identified challenges without a pre-defined coding framework. In the axial coding phase, the two reviewers compared and discussed their initial codes, grouping similar codes into broader categories and identifying relationships between categories. Disagreements were resolved through discussion; when consensus could not be reached, a third reviewer (BC.X) was consulted. In the selective coding phase, core themes were identified through iterative team discussion. Three core operational dimensions emerged consistently across included studies: Deployment (system positioning and configuration), Dispatch (activation and coordination with emergency response), and Delivery (AED transport and transfer to bystanders). These dimensions formed the “3D Framework”.

The final framework was validated through team discussion to ensure all included studies could be mapped onto the identified dimensions. Additionally, six priority areas for future research were identified based on evidence gaps observed across studies: Technical Optimization, System Integration, Deployment Strategy, Human Factors, Clinical Evidence, and Health Economics.

## 3 Results

### 3.1 Literature search and selection results

**Figure 1:**
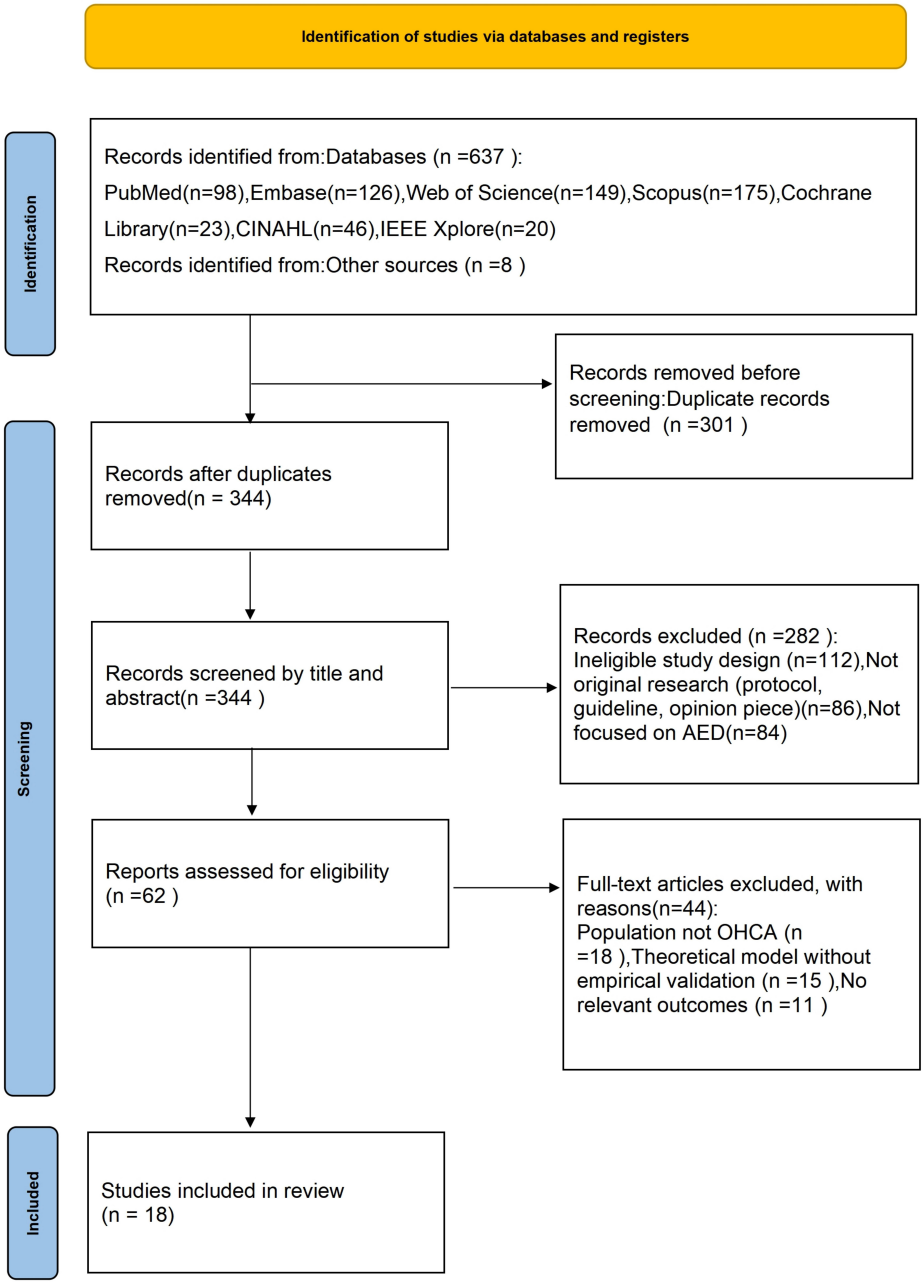
PRISMA flow diagram of the study selection process.

A systematic search of seven electronic databases yielded the following results: PubMed (*n* = 98), Embase (*n* = 126), Web of Science (*n* = 149), Scopus (*n* = 175), Cochrane Library (*n* = 23), CINAHL (*n* = 46), and IEEE Xplore (*n* = 20). Additional records were identified through manual searching, reference list screening, and other supplementary methods (*n* = 8), resulting in a total of 645 records. After removing duplicates using EndNote software, 344 records remained for screening. Following independent title and abstract screening by two reviewers, 62 articles were deemed potentially eligible and advanced to full-text review. After full-text assessment against the predetermined inclusion and exclusion criteria, 18 studies (24–41) were ultimately included in the analysis. The study selection process is illustrated in the PRISMA flow diagram (Figure 1).

### 3.2 Characteristics of included studies

A total of 18 studies (24–41) published between 2016 and 2026 were included in this scoping review. Studies originated from 10 countries, with European nations predominating. Sweden contributed the most studies (*n* = 6) (24, 25, 29, 30, 32, 40), followed by the United States (*n* = 2) (27, 34), Germany (*n* = 2) (28, 33), and China (*n* = 2) (37, 41), while Canada (26), Austria (31), Italy (35), Denmark (36), Thailand (39), and the United Kingdom (38) each contributed one.

**Figure 2:**
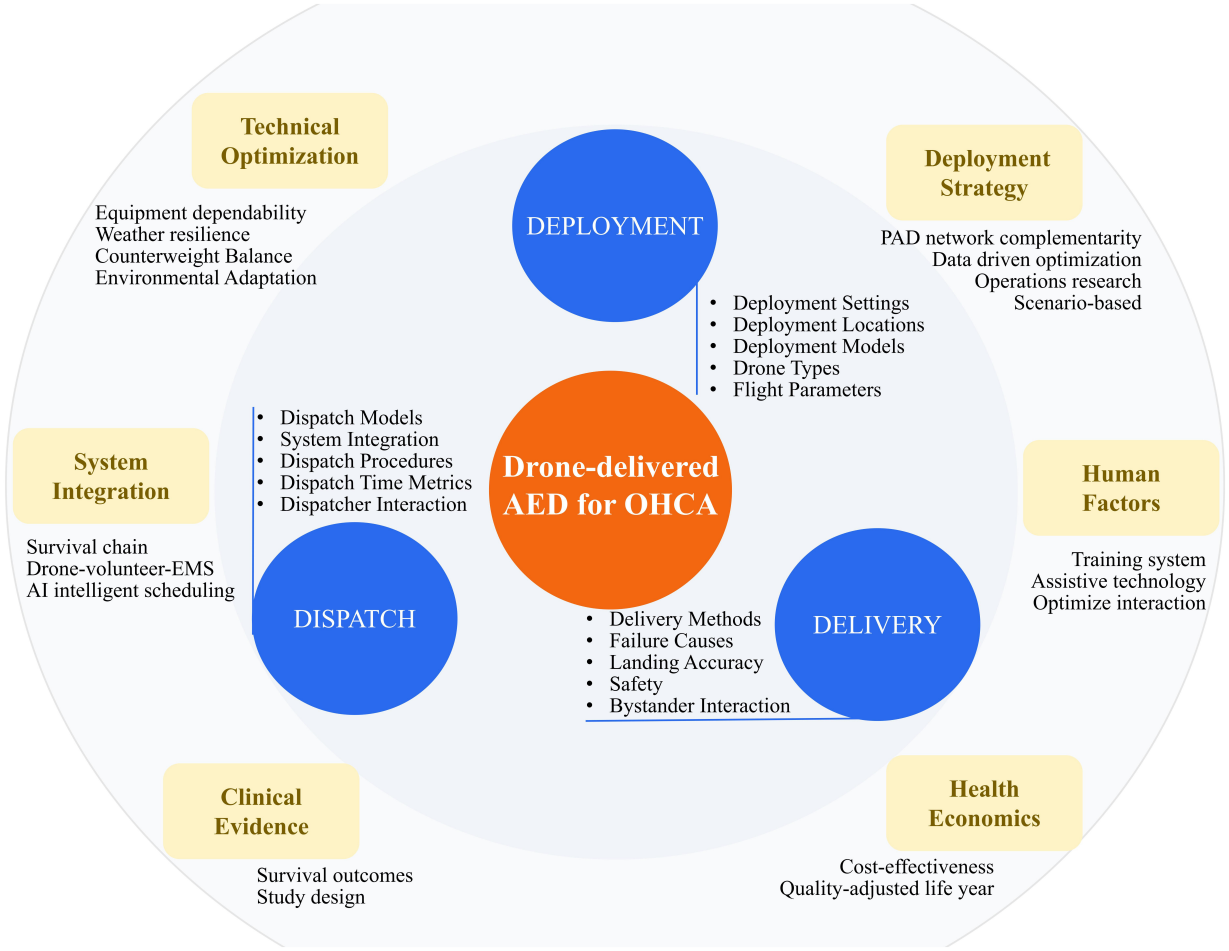
Conceptual framework of drone-delivered AED systems for OHCA response: current evidence domains and future research directions. This figure illustrates the thematic structure of evidence identified in this scoping review and highlights areas for future research. The framework comprises three conceptual layers: (1) Core layer (orange circle): represents the central topic of this review—drone-delivered AED systems for OHCA response; (2) Middle layer (blue circles): represents the three primary evidence domains synthesized in this review: Deployment, Dispatch, and Delivery, with bullet points indicating specific elements examined in the included studies; (3) Outer layer (yellow boxes): represents future research directions and evidence gaps identified through this synthesis, including Technical Optimization, Deployment Strategy, System Integration, Human Factors, Clinical Evidence, and Health Economics. Each research direction is positioned adjacent to the evidence domain(s) it most closely relates to, indicating thematic connections between current evidence and priorities for future investigation.

The included studies were categorized into simulation studies and real-world studies. Simulation studies predominated (*n* = 14, 77.8%) (24–29, 31, 33–35, 37–39, 41), utilizing historical OHCA data or geographic information systems to model response times and coverage. Four real-world studies (22.2%) (30, 32, 36, 40) were identified, comprising one partial report of successful AED delivery to a real OHCA patient (30), two prospective observational studies of suspected OHCA patients (32, 36), and one retrospective analysis of suspected OHCA cases (40). Study settings varied across urban, suburban, and rural environments, with some focusing specifically on remote or prolonged-response-time areas. Detailed study characteristics are summarized in Table 1. The findings were synthesized around three operational dimensions—Deployment, Dispatch, and Delivery—with identified knowledge gaps and future research priorities visualized in Figure 2.

**Table 1:** General characteristics of the included studies (*n* = 18).

Reference	Study setting	Drone model and flight parameters	Deployment and dispatch	AED delivery	Success rate	Drone response time	EMS/ bystander response time	Drone–first arrivals	Main findings
Claesson et al. (24)	Urban and rural	Model: HEIGHT TECH octocopter Speed: 70 km/h Payload: 1.0 kg	Deployment: gis–based modeling for site selection dispatch: no system integration	Parachute release (25 m altitude), low–altitude release (3 m altitude), or direct landing	92.3% (12/13)	–	–	Rural 93%, Urban 32%	Average time advantage of 19 min in rural areas vs. only 1.5 min in urban areas
Claesson et al. (25)	Rural	Model: Swedish Transport Agency–certified octocopter Speed: 50 km/h Payload: 0.76kg	Deployment: fixed station at fire station dispatch: no system integration	Drone landing; bystander unbuckles strap to retrieve AED	100% (18/18)	Launch–to–scene:5 min 21 s	22 min	100%	Drone arrived first in all cases
Cheskes et al. (26)	Remote rural	Model: Sparrow X1000/InDro M210C Speed: ≤ 55 km/h Range: 6.6–8.8 km Payload: 0.49–2.5 kg	Deployment: preset test sites dispatch: simulated system; drone and ambulance dispatched simultaneously	Drone landing; bystander unbuckles strap to retrieve AED	100% (6/6)	Launch–to–scene:5.8–13.0 min	7.5–20.0 min	100%	Drone response time significantly shorter than EMS in rural areas
Rosamond et al. (27)	Urban	Model: DJI Matrice 600 Pro; Max speed: 65 km/h; Range: 238–393 m; Payload: 1.1 kg	Deployment: fixed station at drone launch site dispatch: no system integration	Drone landing; bystander retrieves AED	100% (35/35)	Launch–to–scene:2 min 13 s −2 min 34 s	2 min 56 s−7 min 56 s^a^	–	Drones may enable faster AED acquisition by bystanders
Baumgarten et al. (28)	Rural	Model: Ceptor UAV octocopter Speed: 12.9–47.3 km/h Range: 0.44–9.79 km Payload: 0.78kg	Deployment: fixed stations at emergency centers/fire stations dispatch: simulated system; drone and ambulance dispatched simultaneously	Drone landing; magnetic clamp auto–releases AED	93.8% (46/49)	Alert–to–scene:2 min 22 s −13 min 59 s	–	–	Integration of drone–delivered AED into the OHCA chain of survival is feasible
Schierbeck et al. (29)	Semi–urban	Model: DJI Matrice 600 Pro Speed: 60 km/h Range: 2–3 km Payload: 0.80kg	Deployment: fixed station at drone hangar dispatch: simulated system; drone and ambulance dispatched simultaneously	Hovering at 30 m altitude; parachute–released AED	92% (11/12)	Alert–to–scene:9 min 08 s	9 min 53 s	64%	Drone and EMS response times were comparable in semi–urban areas
Schierbeck et al. (30)	Residential area	Model: Everdrone Speed: 9.4 km/h Range: 673 m	Deployment: fixed station at drone hangar dispatch: EMS–drone integrated dispatch	Winch vertical lowering from 30 m altitude	100% (1/1)	Launch–to–scene:3 min 19 s	6 min 14 s	100%	First documented successful drone–delivered AED defibrillation worldwide; patient discharged with CPC 1
Fischer et al. (31)	Mountainous	Model: AIR8 Medium Lifter Max speed: 90 km/h	Deployment: fixed station at red cross emergency center dispatch: drone–only dispatch	Manual descent to 1 m altitude; AED release	100% (29/29)	Launch–to–scene:5 min 20 s	–	–	Drone–delivered AED deployment and use is feasible in remote mountainous areas
Schierbeck et al. (32)	Semi–urban	Model: DJI Matrice 600 Pro Speed: 48 km/h Median range: 1.8 km Payload: 0.80kg	Deployment: fixed station at drone hangar dispatch: EMS–drone integrated dispatch	Winch vertical lowering from 30 m altitude with audible alert	81% (58/72)	–	–	67%	10.3% of AEDs connected before EMS arrival; 3.4% defibrillation delivered before EMS arrival; drone–delivered AED may reduce time to first defibrillation
Scholz et al. (33)	Rural	Model: Ceptor Transport octocopter Speed: 45 km/h Range: 910–6960 m Payload: 860 g	Deployment: fixed station at drone system base dispatch: no system integration	Drone landing; specialized carrier auto–releases AED	100% (20/20)	Alert–to–release AED:7 min 59 s (daytime) 7 min 26 s (nighttime)	–	–	No significant difference in drone response time between daytime and nighttime deliveries
Starks et al. (34)	Urban public spaces	Model: DJI Matrice 600 Pro Range: 274–366 m Payload: 1.36 kg	Deployment: Drone–AED network dispatch: bystander retrieval guided by T–CPR	Drone landing; bystander retrieves AED	100% (51/51)	–	–	–	CPR–trained individuals retrieved AED from drone faster while maintaining adequate compression quality
van Veelen et al. (35)	Park	Model: MAVTech Q4X; Range: 70–750 m; Average mission speed: ~12 km/h	Deployment: pad site dispatch: dispatcher phone–guided bystander retrieval	Auto–release of parachute–equipped AED case	100% (36/36)	Call–to–shock:2.2 min	12.4 min^1^	–	Drone–delivered AED in urban settings may reduce time to first defibrillation and decrease rescuer fatigue
Jakobsen et al. (36)	Urban	Model: DJI Matrice 600 Pro Speed: ≤ 65 km/h Median range: 1.9 km Payload: 1.0 kg	Deployment: government–designated research area dispatch: integrated into emergency dispatch center system	Winch lowering from 30 m altitude	100% (16/16)	Launch–to–scene:4 min 47 s	3 min 25 s	–	Drone–delivered AED is safe and feasible but dependent on site selection
Jiang et al. (37)	High–rise building	Model: DJI Matrice 350 Altitude: 150 m Range: 2.1 km	Deployment: drone hangar on hospital rooftop dispatch: no system integration	Winch system delivery of AED case	100% (1/1)	Launch–to–scene:5 min 0 s	14 min 3 s	100%	Drone–delivered AED to high–rise residential buildings is feasible and faster than EMS
Smith et al. (38)	–	Model: DJI M300 Median range: 1.17 km	Deployment: preset test sites dispatch: drone–only dispatch	Winch lowering from 10 m altitude; ground–triggered quick–release mechanism	81.8% (9/11)	Launch–to–scene:2 min 19 s	–	–	Interaction barriers among bystanders, dispatchers, and drones after drone arrival represent a significant source of time delay
Srivilaithon et al. (39)	Suburban (university campus)	Model: DJI Matrice 600 Speed: 31.5–33.0 km/h Range: 4009.5–6514.5 m Payload: 0–5.7 kg	Deployment: university campus dispatch: no system integration	Drone landing; bystander unloads AED	97.7% (88/90)	Launch–to–scene:7 min 24 s (AED 2 kg);7 min 32 s (AED 4 kg)	–	–	Increased payload (AED weight) significantly reduces delivery accuracy
Schierbeck et al. (40)	Semi–urban	Model: DJI Matrice 600 Pro Max speed: 60 km/h Max altitude: 65 m	Deployment: not reported dispatch: ems–drone integrated dispatch	Winch lowering from 30 m altitude; dispatcher guides bystander retrieval	100%(123/123)	–	–	–	Dispatcher training may enhance drone time advantage
Suo et al. (41)	Urban	Model: Hexacopter rappelling drone TD9 Max speed: 60.1 km/h Max payload: 9.0 kg Max radius: 6.5 km	Deployment: fixed station at prehospital emergency center dispatch: simulated system; drone and ambulance dispatched simultaneously	Rappelling module rope delivery; auto–release upon ground contact	100% (43/43)	Alert–to–release AED:353 s	597 s	–	The timeliness of AED delivery by drone is significantly better than that of the EMS and PAD

^a^denotes bystander response time data; - indicates the outcome was not reported in the study.

AED, Automated External Defibrillator; GIS, Geographic Information System; EMS, Emergency Medical Services; OHCA, Out-of-Hospital Cardiac Arrest; CPC, Cerebral Performance Category; PAD, Public Access Defibrillation; CPR, Cardiopulmonary Resuscitation; T-CPR, Telephone-assisted Cardiopulmonary Resuscitation. Drone response time definitions varied across studies: Launch-to-scene = time from drone takeoff to arrival at scene; Alert-to-scene = time from dispatch alert to drone arrival at scene; Alert-to-release AED = time from dispatch alert to AED release at scene; Call-to-shock = time from emergency call to first defibrillation. EMS response time refers to the interval from emergency call receipt at the dispatch center to ambulance arrival on scene as reported in each study; Bystander response time includes the time required for bystanders to locate a nearby AED, retrieve it, and return to the scene.

### 3.3 Technical characteristics of drone-delivered AED systems

#### 3.3.1 Drone types and flight parameters

Among the 18 included studies, multirotor drones were the most commonly used type, offering advantages such as vertical takeoff and landing, precise hovering, and visual remote control capabilities. The DJI Matrice 600 Pro was the most widely adopted model, utilized in 7 studies (27, 29, 32, 34, 36, 39, 40). Other commonly used models included octocopters [such as the HEIGHT TECH octocopter (24), Swedish Transport Administration-certified octocopter (25), Ceptor UAV (28), and Ceptor Transport (33)], hexacopters [such as the TD9 rappelling drone (41)], and specialized medical delivery drones [such as Everdrone (30), Sparrow X1000 (26), AIR8 Medium Lifter (31), and MAVTech Q4X (35)].

Flight speeds varied considerably across studies, ranging from 9.4 km/h (30) to 90 km/h (31), with most studies reporting speeds between 30–70 km/h (24–33, 36, 39). Flight distances also showed significant variation, with most drones having a maximum flight radius of 3–10 km. Actual mission flight distances typically ranged from 0.07–3.2 km (25–30, 32–36, 39), with the shortest being 70–750 m (35) and 238–393 m (27) and the longest reaching 6.6–8.8 km (26) and 6.5 km (41). Regarding payload capacity, drones could carry 0.76–9.0 kg, with AED devices and delivery mechanisms typically weighing 0.8–2.5 kg (24–29, 32–34, 36, 39). Notably, Srivilaithon et al. (39) specifically compared the effects of different payloads on flight performance, finding that payload had minimal impact on flight speed (33.0 km/h unloaded vs. 31.5 km/h with 4 kg payload) but significantly affected flight range (6,514.5 m unloaded vs. 4,026.5 m with 4 kg payload).

#### 3.3.2 AED delivery methods

The AED delivery methods employed in the included studies can be categorized into three main types. The first involves the drone landing directly, with bystanders retrieving the AED from the aircraft. This approach was adopted in 7 studies (25–28, 33, 34, 39) and is relatively straightforward but requires suitable landing conditions. The second method uses a winch system to lower the AED vertically from heights typically ranging from 10–30 m, employed in 8 studies (30–32, 36–38, 40, 41). This approach avoids potential risks to ground personnel from drone landing and can be combined with audible alarms to alert bystanders (32). The third method involves parachute-deployed AED release, used in 3 studies (24, 29, 35), with Claesson et al. (24) testing both parachute deployment from 25 m altitude and low-altitude release from 3 m. Additionally, Baumgarten et al. (28) employed a magnetic clamp automatic release mechanism that opens upon landing; Fischer et al. (31) used manual control to lower the drone to 1 m above ground before releasing the AED; and van Veelen et al. (35) utilized GPS-guided automatic release of a parachute-equipped AED container. All delivery methods were designed with safety considerations, effectively minimizing the risk of drone rotors causing injury to personnel on scene. Fischer et al. (31) demonstrated in their simulation study that drones could be manually operated to deliver AEDs at least 3 m away from simulated patients to ensure bystander safety.

#### 3.3.3 Drone-delivered AED system deployment and dispatch

Regarding deployment regions, drone-delivered AED systems have predominantly been deployed in rural, suburban, mountainous, or semi-urban areas where EMS response times are typically longer (24–26, 28, 29, 31–33, 39). Deployment in urban centers has been relatively limited (27, 30, 34–36) due to airspace regulations and landing constraints, reflecting the technology's greater application potential in areas with EMS service gaps or delayed response times. In terms of specific deployment approaches, fixed-site deployment was the most common model, adopted in 10 studies (25, 27–33, 37, 41). Deployment locations included fire stations (25), prehospital emergency centers (28, 31, 41), drone hangars (29, 30, 32), drone system bases (33), drone launch sites (27), and hospital rooftops (37). Claesson et al. (24) constructed GIS models based on historical OHCA hotspot data to optimize site placement strategies. Starks et al. (34) collaborated with local EMS centers and academic institutions to establish a drone-AED network to enhance coverage radius and response efficiency, ensuring drone deployment points existed within 274–366 m of OHCA incident locations. The remaining studies (26, 35, 36, 38, 39) were conducted at preset test sites or government-designated research areas, with deployment location selection influenced by research objectives and aviation regulations.

The dispatch models in included studies demonstrated an evolutionary trend from no system integration to full integration. Early studies primarily consisted of independent tests without dispatch system integration (24, 25, 27, 31, 33, 37–39), mainly aimed at validating the technical feasibility of drone-delivered AEDs. Subsequently, simulated dispatch systems emerged, enabling simultaneous deployment of drones and ambulances (26, 28, 29, 41), providing preliminary evidence for system integration. Real-world studies have achieved various forms of coordinated dispatch between EMS and drone systems. In Swedish studies (30, 32, 40), drone pilots could activate the drone-delivered AED system upon receiving suspected OHCA alerts from the dispatch system, calculating optimal flight paths through route planning software while simultaneously dispatching drones and conventional EMS ambulances for parallel response. The Danish study (36) integrated the drone system into local EMS dispatch center software, automatically activating the drone system when suspected OHCA alerts appeared. Activated drones performed autonomous preflight checks and calculated flight routes based on coordinates and weather conditions. All dispatch procedures required air traffic control approval, with flight control systems monitored by remote pilots and visual recognition systems used to assess landing or drop zone safety (30, 32, 36).

### 3.4 Timeliness evaluation of drone-delivered AEDs

#### 3.4.1 Drone response times

Drone response times reported across included studies varied considerably, influenced by multiple factors including flight distance, flight speed, dispatch procedures, and application settings. Importantly, response time definitions were heterogeneous across studies, making direct comparisons challenging. Outcomes were therefore stratified into four categories based on the time endpoint measured.

Nine studies reported launch-to-scene time, representing the pure flight time from drone takeoff to arrival at the cardiac arrest location. Reported times ranged from 2 min 13 s to 13 min, varying by geographic setting. In urban settings, Rosamond et al. (27) reported 2 min 13 s-2 min 34 s, Smith et al. (38) reported 2 min 19 s, and Jakobsen et al. (36) reported 4 min 47 s. In suburban and residential settings, Schierbeck et al. (30) reported 3 min 19 s in a residential area, while Srivilaithon et al. (39) reported 7 min 24 s−7 min 32 s on a university campus. In rural and remote settings, Claesson et al. (25) reported 5 min 21 s, and Cheskes et al. (26) reported 5.8–13.0 min. Fischer et al. (31) reported 5 min 20 s in a mountainous setting, and Jiang et al. (37) reported 5 min 0 s in a high-rise building scenario.

Two studies reported alert-to-scene time, which includes system activation delay plus flight time. Baumgarten et al. (28) reported times ranging from 2 min 22 s to 13 min 59 s in a rural setting. Schierbeck et al. (29) reported 9 min 08 s in a semi-urban setting. Similarly, Two studies reported alert-to-release AED time, encompassing the full drone operation from dispatch alert to AED release at scene. Scholz et al. (33) reported 7 min 59 s during daytime and 7 min 26 s during nighttime in a rural setting. Suo et al. (41) reported 5 min 53 s in an urban setting. Only one study reported call-to-shock time, representing the most clinically meaningful endpoint measuring the entire chain from emergency call to first defibrillation. Van Veelen et al. (35) reported a call-to-shock time of 2.2 min in a park setting.

#### 3.4.2 Comparison with traditional EMS response times

Seven studies (25, 26, 29, 30, 36, 37, 41) compared drone and traditional EMS response times. As response time definitions varied across studies, direct comparisons should be interpreted with caution. Overall, results showed that drone time advantages were significantly influenced by application setting. In rural and remote settings, drones demonstrated substantial time advantages over traditional EMS. Claesson et al. (25) conducted a simulation study in rural Sweden showing a median drone launch-to-scene time of 5 min 21 s (IQR: 3 min 03 s – 8 min 33 s) compared to 22 min (IQR: 17 min 48 s - 29 min 00 s) for EMS, with drones arriving first in all cases. Similarly, Cheskes et al. (26) showed in a Canadian remote rural simulation that drone launch-to-scene times (5.8–13.0 min) were consistently shorter than EMS response times (7.5–20.0 min), with drones arriving first in all cases. In residential settings, Schierbeck et al. (30) reported that drone launch-to-scene time (3 min 19 s) was approximately half of EMS response time (6 min 14 s), with drones arriving first in all cases. Notably, Jiang et al. (37) demonstrated in a high-rise building scenario that drone launch-to-scene time (5 min 0 s) was substantially shorter than EMS response time (14 min 3 s), with drones arriving first in all cases.

In contrast, drone time advantages in urban and semi-urban settings were less pronounced or even absent, with conflicting findings across studies. Jakobsen et al. (36) found in a Danish urban study that the median drone launch-to-scene time (4 min 47 s) was actually slightly slower than EMS response time (3 min 25 s), suggesting that dispatch efficiency and route planning may limit drone advantages in areas with dense EMS coverage. Similarly, Schierbeck et al. (29) reported in a semi-urban setting that drone alert-to-scene time (9 min 08 s) was only marginally faster than EMS response time (9 min 53 s), with drones arriving first in 64% of cases. However, Suo et al. (41) found in an urban Chinese setting that drone alert-to-release AED time (5 min 53 s) was noticeably shorter than EMS response time (9 min 57 s), indicating that urban drone systems may still offer meaningful time savings depending on local EMS infrastructure and response efficiency. These inconsistent findings suggest that the benefit of drone-delivered AEDs in urban environments may be highly context-dependent, influenced by factors such as EMS density, traffic conditions, and drone dispatch protocols.

#### 3.4.3 Comparison with bystander retrieval of public AEDs

Three studies (27, 35, 41) compared the timeliness of drone-delivered AEDs versus bystander retrieval of public AEDs. Rosamond et al. (27) showed in a U.S. urban study that drone AED delivery took 2 min 13 s to 2 min 34 s, while bystander retrieval time largely depended on the proximity of public AEDs to the OHCA location, ranging from 2 min 56 s to 7 min 56 s. Van Veelen et al. (35) demonstrated in an Italian park study that compared to the 12.4 min required for bystanders to retrieve an AED from a PAD location and return to perform defibrillation, drone-delivered AEDs achieved first shock in an average of only 2.2 min, showing a clear time advantage. Suo et al. (41) also showed that while the distance to public AEDs was shorter than drone flight distance (0.76 km vs. 2.29 km), retrieval time was actually longer (550.1 s vs. 353.0 s).

#### 3.4.4 Factors affecting drone response time

Multiple studies explored factors affecting drone response times. Flight distance was the primary factor, positively correlated with flight time and response time (38). Dispatch activation delay was also an important factor, with Smith et al. (38) finding that 2 min 18 s elapsed from call receipt to drone takeoff. Schierbeck et al. (40) found that following dispatcher education and training, the median time advantage of drones over EMS improved from 1 min 49 s in the non-referral group to 3 min 14 s in the referral group, suggesting that dispatcher training can optimize system performance.

Regarding environmental factors, Scholz et al. (33) compared daytime and nighttime mission performance, finding no significant difference in response times (7 min 59 s vs. 7 min 26 s), indicating that nighttime flights are feasible with appropriate equipment. Concerning payload factors, Srivilaithon et al. (39) evaluated the impact of AED weight on drone response time. The results showed that payload weight had a notable effect on overall response time, with the 2 kg AED group achieving the fastest response time (443.5 s), followed by the 4 kg AED group (451.5 s). This suggests that lighter AED devices may offer advantages in time-critical OHCA scenarios.

### 3.5 Feasibility and safety of drone-delivered AEDs

#### 3.5.1 Delivery success rate and causes of failure

Among the 18 studies (24–41) reporting delivery success rates, overall success rates were high, ranging from 81% to 100%. Twelve studies (25–27, 30, 31, 33–37, 40, 41) reported 100% delivery success rates. The remaining 6 studies reported success rates of 81%-97.7%, including Claesson et al. (24) (92.3%, 12/13), Schierbeck et al. (29) (92%, 11/12), Baumgarten et al. (28) (93.8%, 46/49), Schierbeck et al. (32) (81%, 58/72), Srivilaithon et al. (39) (97.7%, 88/90), and Smith et al. (38) (81.8%, 9/11).

Causes of delivery failure can be categorized into three main domains. Technical factors were the most commonly reported, including parachute system abnormalities leading to mission abort (29), delivery mechanism malfunction (32), intermittent connectivity issues interfering with real-time drone position reporting and triggering automatic return-to-home protocols (38), and other technical failures (28). Environmental factors also played a significant role, with wind speeds exceeding operational limits (28), wind direction affecting landing stability (24), and adverse weather conditions such as snowfall all contributing to delivery failures (28). Additionally, operational factors presented challenges, including remote visual confirmation failure (32), path deviation (32), navigation signal instability, and unsuitable landing sites due to ground obstacles or crowd gatherings (32, 39).

#### 3.5.2 AED landing accuracy

Current studies have not established standard reference values for drone-delivered AED landing accuracy, primarily using “landing distance from patient” as the evaluation metric. Across included studies, common drone-delivered AED accuracy ranged from 3–15 m (28, 29, 31, 32, 35, 36, 39). Schierbeck et al. (32) precisely reported delivery accuracy distribution: 91% of AEDs landed within 15 m of the patient, 64% within 10 m, and 37% within 5 m, demonstrating high flight control and positioning stability. Jakobsen et al. (36) reported that 15 of 16 deliveries landed within 10 m, with 1 within 15 m. Baumgarten et al. (28) showed average landing error not exceeding 8 m, with participants able to reach the AED position within 20 s. Van Veelen et al. (35) reported a mean distance of 4.2 m between drone landing position and bystanders.

Notably, Srivilaithon et al. (39) measured landing deviation under different payload conditions, finding that payload significantly affected delivery accuracy: maximum landing error was 3.35 m with no load, increasing to 28 m with a 2 kg AED payload and reaching 195 m with a 4 kg AED. This finding suggests that payload weight may impact flight control and landing accuracy, warranting attention in high-risk or space-constrained application areas.

#### 3.5.3 Safety

No injuries or serious adverse safety events were reported across included studies, although technical failures (including parachute malfunctions, delivery mechanism failures, and communication interruptions) and landing-related incidents were documented in several studies. Safety assurance was primarily demonstrated in the following aspects: First, safe delivery method design. The three main AED delivery methods in included studies (landing and retrieval, parachute deployment, and winch vertical lowering) all effectively avoided risks of drone rotors causing injury to on-scene personnel. Fischer et al. (31) demonstrated in their simulation study that drones could be manually operated to deliver AEDs at least 3 m from patients to ensure bystander safety. Second, remote monitoring and intelligent recognition. Drone-delivered AED missions were remotely monitored by pilots, with image recognition technology used to assess landing site safety, providing dual technical and human safeguards for mission safety (25, 29, 30, 32, 40). Regarding adverse events, Baumgarten et al. (28) reported one incident of a drone tipping slightly upon landing, which did not affect AED use or cause personnel injury. Schierbeck et al. (29) reported one mission abort due to parachute system abnormality, with the system automatically returning home without causing any risk events. Smith et al. (38) reported two instances where connectivity issues triggered automatic safety features causing drones to return to base, likewise without causing safety incidents.

#### 3.5.4 Impact on time-to-defibrillation and survival

Among the three real-world studies (30, 32, 40), evidence on defibrillation timing and patient outcomes remains limited. In an early prospective study, Schierbeck et al. (32) reported that defibrillation was completed before EMS arrival in 3.4% of 72 suspected OHCA missions. A subsequent retrospective analysis by the same research group (40) demonstrated improved performance, with 42% of patients having AED attached and 6% receiving defibrillation prior to ambulance arrival among 123 cases where drones arrived first. Most notably, Schierbeck et al. (30) documented the world's first successful drone-delivered AED defibrillation, in which the patient received the first shock approximately 45 s before EMS arrival and survived to discharge with CPC score 1. Collectively, these findings provide preliminary real-world evidence that drone-delivered AEDs may shorten time-to-defibrillation and contribute to favorable patient outcomes.

### 3.6 Bystander interaction with drone-delivered AEDs

#### 3.6.1 Bystander AED retrieval time

Whether bystanders can successfully retrieve and correctly use drone-delivered AEDs is a critical factor in determining whether the system can achieve its therapeutic effect. Multiple studies explored this issue from various perspectives. Baumgarten et al. (28) reported that participants could reach and retrieve the AED within 20 s of landing. Smith et al. (38) provided detailed analysis of time consumption during human-drone interaction, finding that 4 min 10 s elapsed from drone arrival to AED attachment, with an additional 25 s from attachment to shock initiation. Only 16 s was spent leaving the patient to retrieve the AED, while hands-off CPR time was 2 min 32 s. Moreover, Starks et al. (34) compared the performance of bystanders who had recently received CPR training versus those who had not, finding median times from AED retrieval from the drone to shock delivery of 1 min 55 s and 2 min 10 s respectively, a difference of 15 s favoring the trained group. Fischer et al. (31) showed that the mean time from AED delivery to first shock was significantly shorter for healthcare providers (79 s) than for laypersons (140 s), a difference of approximately 1 min. These findings support the necessity of strengthening public first aid skills training alongside the promotion of drone-delivered AED systems.

#### 3.6.2 Technical aids to assist bystanders

To help bystanders locate and retrieve AEDs, included studies employed various technical aids. Schierbeck et al. (32, 40) combined winch-deployed AEDs with audible alarm devices to alert ground personnel to collect them. Van Veelen et al. (35) and other studies used dispatcher telephone guidance to direct bystanders through the entire process of AED retrieval and use. Starks et al. (34) conducted their study within a T-CPR (telephone-assisted CPR) framework, with dispatchers simultaneously guiding bystanders in performing CPR and retrieving AEDs from drones.

## 4 Discussion

Deployment strategy for drone-delivered AED systems directly influences coverage and response efficiency. Evidence from studies included in this review demonstrated that current deployment models range from single-station proof-of-concept implementations to multi-site regional coverage networks, with deployment settings spanning urban, suburban, rural, and remote areas. Included studies showed considerable variation in drone base locations, including fire stations, hospitals, emergency dispatch centers, and dedicated drone stations, reflecting the absence of standardized deployment guidelines. Previous literature suggests that optimizing deployment requires balancing multiple interrelated factors. First, historical OHCA data should guide identification of high-incidence hotspots for prioritized resource allocation; Schierbeck et al. (42) demonstrated through GIS analysis that 61 drone systems in Sweden could cover 58.2% of OHCA patients with 5 min average time savings, expandable to nationwide coverage with 2,408 systems. Second, deployment density must balance cost against coverage—excessive density increases operational expenses while insufficient density risks failing to reach patients within the critical intervention window. Mathematical modeling and operations research approaches, including Maximum Covering Models, Location Set Covering Problems, P-Median Problems, and related methods (43, 44), can inform these decisions. Finally, practical constraints including airspace conditions, meteorological factors, and maintenance accessibility must be considered, as external research indicates that environmental and airspace issues account for 79% of regulatory barriers to deployment (44). Effective planning therefore requires early coordination with aviation authorities to balance technical feasibility with regulatory compliance.

The core value of drone-delivered AEDs lies in shortening the time window for early defibrillation within the OHCA chain of survival (16). Studies included in this review provided evidence regarding dispatch procedures and integration approaches. Included studies demonstrated that dispatch models varied from manual activation by emergency dispatchers to semi-automated systems triggered upon OHCA recognition. Several included studies reported dispatch delays ranging from seconds to minutes, attributable to dispatcher decision-making time, system activation procedures, and communication processes between dispatch centers and drone operators (34, 41). Findings from included studies also indicated that real-time communication between dispatchers and bystanders during drone flight, including telephone-guided CPR instructions and voice prompts upon drone arrival, facilitated bystander interaction with the delivered AED (34). However, realizing the full advantage of drone-delivered AEDs depends on seamless integration with other chain of survival components (45). External literature suggests that if drone systems operate as isolated modules disconnected from existing prehospital dispatch systems, issues such as activation delays, information gaps, or redundant resource allocation may arise, ultimately undermining time efficiency (46). From a chain of survival optimization perspective, broader contextual evidence indicates that ideal integration should span the entire process from early recognition to early defibrillation (30). Upon identifying suspected OHCA through standardized questioning, dispatch centers should simultaneously trigger drone deployment and EMS dispatch with a single action, avoiding secondary decision delays (34). To achieve closed-loop integration within a “human-machine-network” collaborative framework, transforming drones from isolated technical tools into intelligent nodes embedded within the chain of survival (47), external researchers recommend that prehospital dispatch platforms integrate with public AED network databases and community first-responder volunteer systems. This would enable simultaneous mobilization of nearby registered volunteers alongside drone deployment, creating a “drone-volunteer-EMS” multi-response model that maximizes defibrillation time reduction and bystander intervention likelihood (48). Additionally, AI-based dynamic dispatch algorithms that automatically calculate optimal response plans based on patient location, real-time drone status, meteorological conditions, and airspace restrictions may further enhance drone efficiency (49).

Advancing drone-delivered AED integration into the chain of survival requires addressing technical and operational challenges identified in the included studies. Evidence from included studies identified several key factors affecting drone flight performance and mission success. First, key factors that have been identified as potentially affecting drone flight require optimization. At the technical level, equipment reliability issues including parachute system malfunctions, delivery mechanism failures, and communication interruptions must be resolved (50). Environmental factors represent another major challenge; a winter study demonstrated that adverse weather conditions such as strong winds, low temperatures, and snowfall not only compromise flight safety and cause mission interruptions but also reduce battery performance and flight endurance (26), making enhanced drone resilience to extreme weather essential for practical implementation and expanded application scenarios. Additionally, spatial environmental factors including complex terrain, building obstruction, and GPS signal interference challenge flight stability and positioning accuracy (51). Furthermore, human factors including remote visual confirmation failures, inappropriate landing site selection, and pre-flight preparation delays may affect drone-delivered AED mission execution (28, 32). Addressing these challenges requires advancing multi-sensor fusion, real-time meteorological monitoring, adaptive route planning, and establishing dynamic risk assessment-based mission decision mechanisms as prerequisites for successful emergency mission execution (46).

Second, evidence from included studies demonstrated the inherent trade-off between payload capacity and flight performance in AED delivery system design. Included studies reported that heavier payloads increased flight time and energy consumption and caused greater landing deviation, prolonging the time for bystanders to locate the AED (52). Studies found that response times for 2 kg versus 4 kg AED payload missions can differ by tens of seconds—for OHCA, an extremely time-sensitive emergency, even delays of tens of seconds may substantially reduce survival probability (34, 39). Conversely, larger drones are more prone to positioning errors in complex terrain or confined spaces, and strong downwash from larger rotors may cause discomfort to ground personnel (53). External literature suggests that this issue can be addressed from two perspectives: optimizing drone platform design to improve payload capacity and endurance, enabling stable operation under broader conditions (54); and promoting lightweight AED design that reduces weight and volume while preserving defibrillation function, developing Personal Portable AEDs specifically optimized for drone delivery scenarios (55).

Finally, regarding delivery methods, studies included in this review examined three primary approaches: direct landing, winch lowering, and parachute drop (25, 29, 32, 34–36). Direct landing offers relatively simple operation with precise AED positioning for easy bystander retrieval, but requires adequate open space for safe landing and may be limited in densely built or complex terrain areas (25, 34). Winch lowering allows the drone to hover above the target and precisely lower the AED to the ground, requiring less landing space, but increases system complexity and demands higher hover stability and favorable wind conditions (32, 36). Parachute drop enables AED release during flight without requiring drone landing, suitable for difficult-to-access locations, but delivery accuracy is significantly affected by wind, potentially prolonging bystander search time (29, 35). Based on findings from included studies, delivery method selection likely needs to be tailored to application scenario characteristics, and future research should further compare the performance of different delivery methods across various scenarios to inform system design and emergency response decisions.

Studies included in this review consistently demonstrated that drone-delivered AEDs exhibit the most pronounced time advantages in rural and remote areas. This can be attributed to the inherent limitations of conventional EMS vehicles, which are constrained by road infrastructure, traffic conditions, and terrain, making geographic accessibility a core barrier to effective OHCA response in these regions (46). Drones, by contrast, can fly directly to target locations via the shortest aerial path, bypassing these ground-level constraints. This technological advantage positions drone-delivered AEDs as a promising solution for bridging emergency service gaps in underserved areas, supporting strategies that prioritize deployment in regions with prolonged EMS response times and inadequate public AED coverage (55). Ultimately, the time saved may directly translate into improved patient survival (48). In contrast, the time advantages of drone-delivered AEDs in urban areas are relatively limited, with some studies reporting response times comparable to or slightly slower than conventional EMS (36). This phenomenon reflects multiple factors: well-developed EMS networks with high station density result in shorter baseline response times (56); stricter airspace regulations and flight restrictions over high-rise buildings increase operational complexity (14); prevalent no-fly zones may force drones to take detours (14); and limited suitable landing sites constrained by obstacles and crowds may prolong AED retrieval time (54). Nevertheless, this does not preclude urban deployment. Boutilier et al. (57) found that deploying 100 drones across 81 stations in Toronto could achieve AED delivery 3 min faster than 911-dispatched EMS. Accordingly, urban deployment strategies should emphasize data-driven optimization based on historical OHCA incidence and population density distribution, positioning drones as a precision supplement to existing emergency systems rather than a comprehensive replacement.

Furthermore, drone-delivered AEDs and PAD networks represent two distinct yet complementary early defibrillation strategies. Evidence from included studies comparing drone-delivered AEDs with PAD networks demonstrated that drones could deliver AEDs faster than bystander retrieval from public AED locations in many scenarios, particularly when existing PAD coverage was sparse or when AED locations were unfamiliar to bystanders. The earlier literature provides context for understanding these findings. The core limitations of PAD networks lie in their uneven geographic distribution and restricted accessibility (51). Public AEDs are typically deployed in high-traffic locations such as airports, shopping centers, and sports venues, yet approximately 70% of OHCAs occur in residential settings where AED coverage is often lacking (47). Even when public AEDs are nearby, bystanders may fail to retrieve them due to unfamiliarity with their location, inability to access the premises, or reluctance to leave the patient (47). Drone-delivered AEDs address these barriers by actively transporting devices to the scene, eliminating the need for bystanders to leave the patient and potentially reducing no-flow time and time-to-first-defibrillation (48). Based on evidence from both included studies and previous studies, drone-delivered AEDs and PAD networks should be viewed as complementary rather than competing approaches. In urban commercial areas with dense PAD coverage, existing networks may already provide adequate protection, whereas drone delivery systems offer greater value in underserved residential, rural, and remote areas. Future emergency response planning should consider integrating both strategies, selecting optimal AED configurations based on regional characteristics.

It is important to note that the current evidence base is predominantly composed of simulation studies, with real-world implementation data still emerging. Therefore, while technical feasibility appears promising, the clinical effectiveness of drone-delivered AEDs in improving patient outcomes remains to be established through rigorous prospective studies (51). To advance drone-delivered AEDs from technical validation to evidence-based practice, future research should prioritize the following methodological directions. First, systematic collection of clinical core outcome set data from real-world OHCA patients is essential (58). Meanwhile, rigorous comparative studies should be designed to evaluate the incremental benefits of drone-delivered AEDs over conventional emergency response models. Given the ethical and implementation challenges of randomized controlled trials in the OHCA context, alternative designs such as before-after comparisons, stepped-wedge designs, or quasi-experimental studies comparing drone-covered versus non-covered areas warrant consideration. Second, future research should develop training programs that incorporate drone-delivered AED content into professional prehospital personnel education and public first-aid training systems, exploring strategies to optimize human-machine interaction, lower operational barriers, and increase technology acceptance to achieve synergistic effects (47). Third, conducting cost-effectiveness analyses to evaluate the economic value of drone-delivered AED systems deserves attention, requiring comprehensive consideration of indicators including system construction costs, operational and maintenance expenses, and cost per quality-adjusted life year saved, thereby providing economic evidence for public health policy decisions and resource allocation (48).

This scoping review has several limitations. First, this review was limited to studies published in English and Chinese. Although these represent the predominant languages in this field, relevant studies published in other languages may have been missed, potentially introducing language bias. Second, considerable heterogeneity existed among included studies in terms of study designs, settings, and outcome definitions (particularly response time metrics), precluding direct comparison and quantitative meta-analysis. Third, the current evidence base has notable gaps: most studies employed simulation designs with limited real-world evidence from actual OHCA scenarios, and included studies were predominantly from European and North American countries, limiting generalizability to other geographic and healthcare contexts.

## 5 Conclusions

Current evidence, predominantly from simulation studies, suggests that drone-delivered AED systems demonstrate preliminary technical feasibility and potential time advantages, particularly in rural and underserved areas. However, real-world clinical evidence remains limited, and findings should be interpreted with caution given language restrictions, study heterogeneity, and limited geographic and socioeconomic diversity of included studies. Further research is needed to validate these preliminary findings. Future research should prioritize technical optimization, system integration, clinical evidence generation, human factors, deployment strategy refinement, and health economic evaluation to facilitate translation from pilot projects to routine clinical implementation.

## Data Availability

The original contributions presented in the study are included in the article, further inquiries can be directed to the corresponding authors.
