# Supplementary material for: Deployment, dispatch, and delivery: a scoping review of drone-delivered AED for out-of-hospital cardiac arrest
Source: Front Public Health. 2026 May 29;14:1839209. doi: 10.3389/fpubh.2026.1839209 (PMC13260238; doi:10.3389/fpubh.2026.1839209)
Supplement: Supplementary file 1 [file Supplementary_file_1.docx]

Supplementary Material

# Preferred Reporting Items for Systematic reviews and Meta-Analyses extension for Scoping Reviews (PRISMA-ScR) Checklist

| **SECTION** | **ITEM** | **PRISMA-ScR CHECKLIST ITEM** | **REPORTED ON SECTION #** |
| --- | --- | --- | --- |
| **TITLE** | | | |
| Title | 1 | Identify the report as a scoping review. | Title |
| **ABSTRACT** | | | |
| Structured summary | 2 | Provide a structured summary that includes (as applicable): background, objectives, eligibility criteria, sources of evidence, charting methods, results, and conclusions that relate to the review questions and objectives. | Abstract |
| **INTRODUCTION** | | | |
| Rationale | 3 | Describe the rationale for the review in the context of what is already known. Explain why the review questions/objectives lend themselves to a scoping review approach. | Introduction |
| Objectives | 4 | Provide an explicit statement of the questions and objectives being addressed with reference to their key elements (e.g., population or participants, concepts, and context) or other relevant key elements used to conceptualize the review questions and/or objectives. | Introduction |
| **METHODS** | | | |
| Protocol and registration | 5 | Indicate whether a review protocol exists; state if and where it can be accessed (e.g., a Web address); and if available, provide registration information, including the registration number. | Methods |
| Eligibility criteria | 6 | Specify characteristics of the sources of evidence used as eligibility criteria (e.g., years considered, language, and publication status), and provide a rationale. | Eligibility Criteria |
| Information sources* | 7 | Describe all information sources in the search (e.g., databases with dates of coverage and contact with authors to identify additional sources), as well as the date the most recent search was executed. | Search Strategy |
| Search | 8 | Present the full electronic search strategy for at least 1 database, including any limits used, such that it could be repeated. | Search Strategy |
| Selection of sources of evidence† | 9 | State the process for selecting sources of evidence (i.e., screening and eligibility) included in the scoping review. | Eligibility Criteria  Study Selection |
| Data charting process‡ | 10 | Describe the methods of charting data from the included sources of evidence (e.g., calibrated forms or forms that have been tested by the team before their use, and whether data charting was done independently or in duplicate) and any processes for obtaining and confirming data from investigators. | Data Extraction |
| Data items | 11 | List and define all variables for which data were sought and any assumptions and simplifications made. | Data Extraction |
| Critical appraisal of individual sources of evidence§ | 12 | If done, provide a rationale for conducting a critical appraisal of included sources of evidence; describe the methods used and how this information was used in any data synthesis (if appropriate). | Not applicable |
| Synthesis of results | 13 | Describe the methods of handling and summarizing the data that were charted. | Data Synthesis |
| **RESULTS** | | | |
| Selection of sources of evidence | 14 | Give numbers of sources of evidence screened, assessed for eligibility, and included in the review, with reasons for exclusions at each stage, ideally using a flow diagram. | Results，Figure1 |
| Characteristics of sources of evidence | 15 | For each source of evidence, present characteristics for which data were charted and provide the citations. | Results,Table1 |
| Critical appraisal within sources of evidence | 16 | If done, present data on critical appraisal of included sources of evidence (see item 12). | Not applicable |
| Results of individual sources of evidence | 17 | For each included source of evidence, present the relevant data that were charted that relate to the review questions and objectives. | Table1 |
| Synthesis of results | 18 | Summarize and/or present the charting results as they relate to the review questions and objectives. | Results,  Figure2 |
| **DISCUSSION** | | | |
| Summary of evidence | 19 | Summarize the main results (including an overview of concepts, themes, and types of evidence available), link to the review questions and objectives, and consider the relevance to key groups. | Discussion |
| Limitations | 20 | Discuss the limitations of the scoping review process. | Discussion |
| Conclusions | 21 | Provide a general interpretation of the results with respect to the review questions and objectives, as well as potential implications and/or next steps. | Conclusions |
| **FUNDING** | | | |
| Funding | 22 | Describe sources of funding for the included sources of evidence, as well as sources of funding for the scoping review. Describe the role of the funders of the scoping review. | Funding |

# Search strategies and results for each database

| **Databases** | **Search strategy** | **Results** |
| --- | --- | --- |
| Pubmed | (("Unmanned Aerial Devices"[MeSH Terms]) OR (drone*[Title/Abstract]) OR (unmanned aerial vehicle*[Title/Abstract]) OR (UAV[Title/Abstract]) OR (unmanned aircraft system*[Title/Abstract]) OR (UAS[Title/Abstract]) OR (remotely piloted aircraft*[Title/Abstract])) AND (("Defibrillators"[MeSH Terms]) OR (automated external defibrillator*[Title/Abstract]) OR (automatic external defibrillator*[Title/Abstract]) OR (AED[Title/Abstract]) OR (defibrillator*[Title/Abstract]) OR (defibrillation[Title/Abstract])) AND (("Out-of-Hospital Cardiac Arrest"[MeSH Terms]) OR (out-of-hospital cardiac arrest*[Title/Abstract]) OR (OHCA[Title/Abstract]) OR (out of hospital cardiac arrest[Title/Abstract]) OR (sudden cardiac arrest*[Title/Abstract]) OR (sudden cardiac death[Title/Abstract]) OR (cardiac arrest[Title/Abstract])) | 98 |
| Web of science | TS=(drone* OR "unmanned aerial vehicle*" OR UAV OR UAVs OR "unmanned aircraft system*" OR UAS OR "remotely piloted aircraft*" OR multirotor* OR quadcopter*)  AND  TS=("automated external defibrillator*" OR "automatic external defibrillator*" OR AED OR AEDs OR defibrillator* OR defibrillation)  AND  TS=("out-of-hospital cardiac arrest*" OR OHCA OR "out of hospital cardiac arrest*" OR "sudden cardiac arrest*" OR "sudden cardiac death*" OR "cardiac arrest*") | 149 |
| Embase | 1. exp drone/ or (drone* or "unmanned aerial vehicle*" or UAV or UAVs or "unmanned aircraft system*" or UAS or "remotely piloted aircraft*" or multirotor* or quadcopter*).ti,ab,kw.  2. exp automated external defibrillator/ or exp defibrillator/ or ("automated external defibrillator*" or "automatic external defibrillator*" or AED or AEDs or defibrillator* or defibrillation).ti,ab,kw.  3. exp out of hospital cardiac arrest/ or exp heart arrest/ or ("out-of-hospital cardiac arrest*" or OHCA or "out of hospital cardiac arrest*" or "sudden cardiac arrest*" or "sudden cardiac death*" or "cardiac arrest*").ti,ab,kw.  4. 1 and 2 and 3 | 126 |
| Cochrane Library | #1 (drone* OR "unmanned aerial vehicle*" OR UAV OR UAVs OR "unmanned aircraft system*" OR UAS OR "remotely piloted aircraft*" OR multirotor* OR quadcopter*):ti,ab,kw  #2 ("automated external defibrillator*" OR "automatic external defibrillator*" OR AED OR AEDs OR defibrillator* OR defibrillation):ti,ab,kw  #3 ("out-of-hospital cardiac arrest*" OR OHCA OR "out of hospital cardiac arrest*" OR "sudden cardiac arrest*" OR "sudden cardiac death*" OR "cardiac arrest*"):ti,ab,kw  #4 #1 AND #2 AND #3 | 23 |
| Scopus | TITLE-ABS-KEY(drone* OR "unmanned aerial vehicle*" OR UAV OR UAVs OR "unmanned aircraft system*" OR UAS OR "remotely piloted aircraft*" OR multirotor* OR quadcopter*)  AND  TITLE-ABS-KEY("automated external defibrillator*" OR "automatic external defibrillator*" OR AED OR AEDs OR defibrillator* OR defibrillation)  AND  TITLE-ABS-KEY("out-of-hospital cardiac arrest*" OR OHCA OR "out of hospital cardiac arrest*" OR "sudden cardiac arrest*" OR "sudden cardiac death*" OR "cardiac arrest*") | 175 |
| CINAHL | S1 (MH "Unmanned Aerial Vehicles") OR TI(drone* OR "unmanned aerial vehicle*" OR UAV OR UAVs OR "unmanned aircraft system*" OR UAS OR "remotely piloted aircraft*" OR multirotor* OR quadcopter*) OR AB(drone* OR "unmanned aerial vehicle*" OR UAV OR UAVs OR "unmanned aircraft system*" OR UAS OR "remotely piloted aircraft*" OR multirotor* OR quadcopter*)  S2 (MH "Defibrillators") OR TI("automated external defibrillator*" OR "automatic external defibrillator*" OR AED OR AEDs OR defibrillator* OR defibrillation) OR AB("automated external defibrillator*" OR "automatic external defibrillator*" OR AED OR AEDs OR defibrillator* OR defibrillation)  S3 (MH "Heart Arrest") OR TI("out-of-hospital cardiac arrest*" OR OHCA OR "out of hospital cardiac arrest*" OR "sudden cardiac arrest*" OR "sudden cardiac death*" OR "cardiac arrest*") OR AB("out-of-hospital cardiac arrest*" OR OHCA OR "out of hospital cardiac arrest*" OR "sudden cardiac arrest*" OR "sudden cardiac death*" OR "cardiac arrest*")  S4 S1 AND S2 AND S3 | 46 |
| IEEE Xplore | ("All Metadata":drone* OR "All Metadata":"unmanned aerial vehicle" OR "All Metadata":UAV OR "All Metadata":"unmanned aircraft system" OR "All Metadata":UAS OR "All Metadata":"remotely piloted aircraft" OR "All Metadata":quadcopter*)  AND  ("All Metadata":"automated external defibrillator" OR "All Metadata":"automatic external defibrillator" OR "All Metadata":AED OR "All Metadata":defibrillator* OR "All Metadata":defibrillation)  AND  ("All Metadata":"out-of-hospital cardiac arrest" OR "All Metadata":OHCA OR "All Metadata":"cardiac arrest" OR "All Metadata":"sudden cardiac arrest" OR "All Metadata":"sudden cardiac death") | 20 |
